# Discovery of Afifi, the shallowest and southernmost brine pool reported in the Red Sea

**DOI:** 10.1038/s41598-020-57416-w

**Published:** 2020-01-22

**Authors:** Carlos M. Duarte, Anders Røstad, Grégoire Michoud, Alan Barozzi, Giuseppe Merlino, Antonio Delgado-Huertas, Brian C. Hession, Francis L. Mallon, Abdulakader M. Afifi, Daniele Daffonchio

**Affiliations:** 1 Red Sea Research Center (RSRC) and Computational Bioscience Research Center, King Abdullah University of Science and Technology (KAUST), Thuwal, 23955-6900 Saudi Arabia; 2 Red Sea Research Center (RSRC) and Biological and Environmental Science and Engineering Division (BESE), King Abdullah University of Science and Technology (KAUST), Thuwal, 23955-6900 Saudi Arabia; 3 Instituto Andaluz de Ciencias de la Tierra, CSIC-UGR, Avda. de las Palmeras 4, 18100 Armilla, Spain; 4 Coastal and Marine Resources Core Lab (CMOR), King Abdullah University of Science and Technology (KAUST), Thuwal, 23955-6900 Saudi Arabia; 5 Ali I. Al-Naimi Petroleum Engineering Research Center (ANPERC), Physical Science and Engineering Division (PSE), King Abdullah University of Science and Technology (KAUST), Thuwal, 23955-6900 Saudi Arabia

**Keywords:** Marine biology, Marine chemistry

## Abstract

The previously uncharted Afifi brine pool was discovered in the eastern shelf of the southern Red Sea. It is the shallowest brine basin yet reported in the Red Sea (depth range: 353.0 to 400.5 m). It presents a highly saline (228 g/L), thalassohaline, cold (23.3 °C), anoxic brine, inhabited by the bacterial classes KB1, Bacteroidia and Clostridia and the archaeal classes Methanobacteria and Deep Sea Euryarcheota Group. Functional assignments deduced from the taxonomy indicate methanogenesis and sulfur respiration to be important metabolic processes in this environment. The Afifi brine was remarkably enriched in dissolved inorganic carbon due to microbial respiration and in dissolved nitrogen, derived from anammox processes and denitrification, according to high δ^15^N values (+6.88‰, AIR). The Afifi brine show a linear increase in δ^18^O and δD relative to seawater that differs from the others Red Sea brine pools, indicating a non-hydrothermal origin, compatible with enrichment in evaporitic environments. Afifi brine was probably formed by venting of fossil connate waters from the evaporitic sediments beneath the seafloor, with a possible contribution from the dehydration of gypsum to anhydrite. Such origin is unique among the known Red Sea brine pools.

## Introduction

Submarine brine pools are bodies of water with elevated salinity that form lake-like structures on the seafloor. The origin of these brines has been attributed to dissolution of evaporitic deposits in sedimentary layers below the seafloor, or from dissolution of salt flows^1–3^. The dense brines flow from vents gravitationally down the seafloor topography and may accumulate in bathymetric depressions and cuvettes. Hot (hydrothermal) brine pools and vents generally occur along mid-ocean ridges throughout the world, including the central Red Sea. Cold brine pools and vents, often associated with hydrocarbon seeps, occur in a variety of tectonic settings around the world, including the Mediterranean Sea and the Gulf of Mexico^4,5^, and have also been reported from the eastern margin of the Red Sea^6^.

The Red Sea resulted from rifting and separation of the Arabian Plate from the African Plate, starting around 30 Ma ago^7^. Seafloor spreading started around 15 Ma ago coincident with westward propagation of the Sheba ridge into the Gulf of Aden, and the activation of the Aqaba/Levant transform^8^. The continental shelves in the Red Sea are underlain by a thick section of Neogene sediments which include Middle - Late Miocene evaporites. These evaporites consist of a massive salt layer as well as layered anhydrite^9^. The salt was mobilized into a variety of diapiric structures, and its original thickness was estimated at 1.5–2 kilometers^10^. The salt also forms salt flows on the seafloor^11^, partly covering the oceanic crust in the southern Red Sea, and almost completely covering it in the north.

First discovered in 1965^12^, the hot deep-sea brine pools of the Red Sea have received much attention, including geochemical^13,14^, hydrographical^15,16^ and microbiological^17^ characterization, as well as modelling of the formation of these features^18^. These studies have established that the hot dense brines formed from dissolution of salt by hydrothermal fluids circulating through fractured oceanic crust, which then settled within enclosed bathymetric depressions. The brine pools are in dynamic equilibrium, maintained by input of brines flowing into the depressions being compensated by overspill and subsequent loss of brines^19^. The hot brine pools are typically anoxic, hypersaline (up to 270‰), and tend to be warmer, by up 46 °C, than the overlying seawater^16^ (Table S1). The hot brine pools in the Red Sea occur along the mid-ocean ridge at depths exceeding 1,000 m (Table S1). The hot brine pools in the Red Sea have received scientific interest not only for their microbiological and biogeochemical aspects, but also because they are underlain by sulfide - rich muds that contain a significant resource of metals^20^.

**Figure 1:**
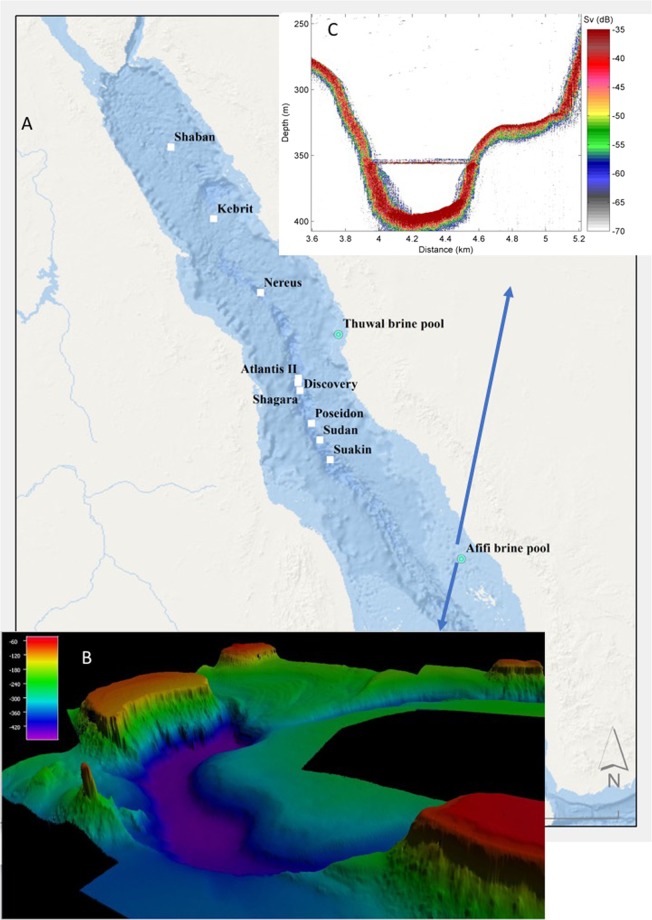
(**A**) Map showing the location of the Afifi brine pole relative to other brine pools in the Red Sea. Hot and cold brine pools are shown is white squares and green dots, respectively. (**B**) Bathymetry of the Afifi brine pool based on multibeam echosounder surveys and (**C**) cross section of the brine as recorded on the echosounder, where the brine surface, seen as a thin line, has a strong sound reflection.

In contrast to the hot brine pools within the axial deeps along the Red Sea axis, the Thuwal brine pool was discovered^6^ at a distance of only 20 kilometers from the Arabian shoreline (Fig. 1). Its depth (840 meters), temperature (21.7 °C) and salinity (74 psu) were the lowest among the Red Sea brines. Furthermore, it is located adjacent to a sunken reef 100 kilometers across from the axial valley of the Red Sea, which indicates a geologic setting unrelated to hydrothermal activity at mid-ocean ridges.

Here we report the discovery of the Afifi brine pool, which is named after one of our co-authors for his dedication to scientific research on the Red Sea. It lies at a depth of about 350 m just north of the Farasan Islands (Fig. 1), and is the southernmost and shallowest of all brine pools reported in the Red Sea. Yet, it ranks amongst the saltiest of all known brine pools. We characterize its location, size, oceanographic setting and general geochemical conditions, as well as the microbial communities associated with this brine pool.

## Results and Discussion

### Discovery of afifi brine

The Afifi brine pool was discovered by observing a perfectly flat water column reflection from the top of the brine surface detected by the Simrad EK60 echosounder (Fig. 1), similar to that characteristic of brine pools elsewhere. It is found ~415 km southern than the previous discovered brines and located far from the Red Sea central axis where the other basins are found (17°33.6′N, 41°27.6′E), with the exception of the Thuwal Seeps (Table S1). The top of the brine pool lies at a depth of about 353 meters and is much shallower than the other brine pools in the Red Sea that are found below 1500 m up to 2850 m with the exception of the Thuwal Seeps located around 816 m (Table S1). Examination of the bathymetric setting showed that the Afifi brine pool sits in a curved 3 km long bathymetric depression bounded by shallow carbonate platforms and salt flows. (Fig. 1). The maximum depth of the seafloor under the brine pool is 400.5 m, with the brine interface located 353 m below the sea level, resulting in an averaged brine pool thickness of 29.2 m (from seafloor to brine-overlaying water interface), a surface area of 1.08 km^2^ and 0.032 km^3^ of brine volume.

### Physicochemical properties of the Afifi brine

**Figure 2:**
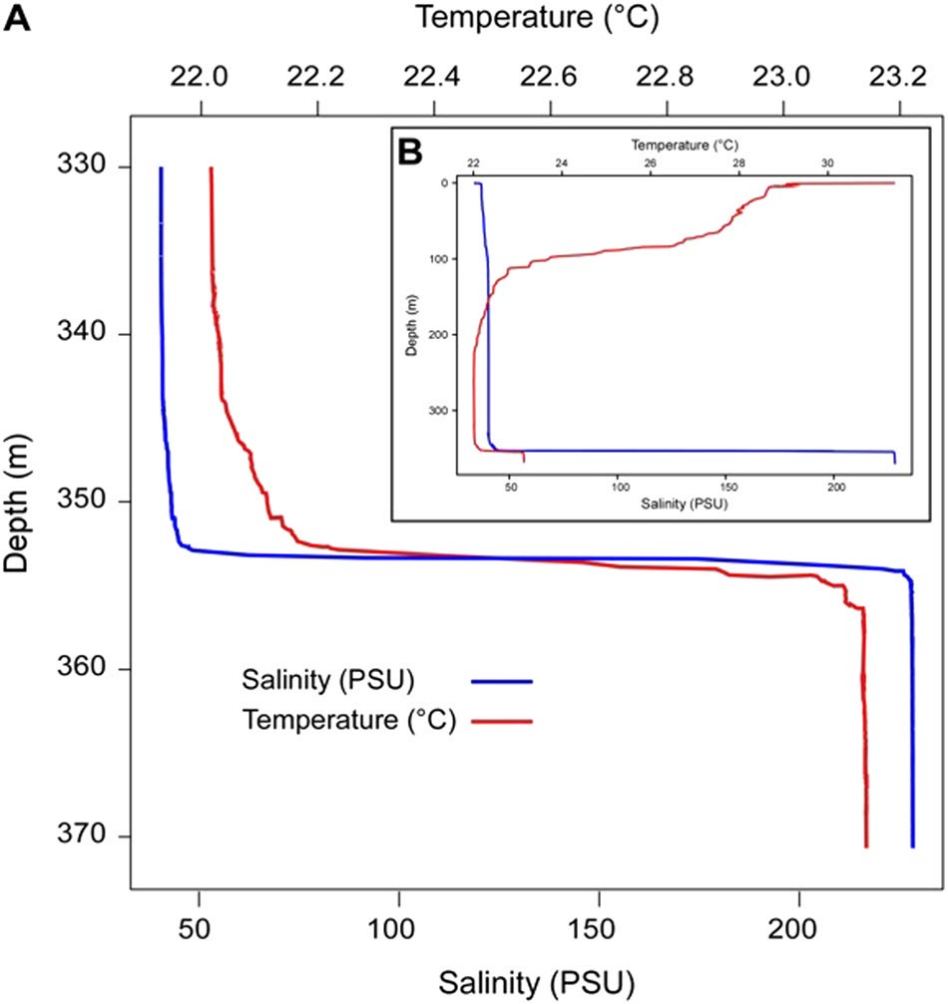
Temperature and depth profiles from the Afifi brine pool along the brine seawater interface (**A**) and all the water column (**B**).

The salinity of the Afifi brine is 228 PSU, resulting in a sharp increase from 41 PSU measured in the overlying oxic water column above 353 m (Fig. 2A,B). It is consistent with elevated salinity levels registered in the other Red Sea brine basins^5,21^. The salinity of the brine is mainly contributed by NaCl (Table 1) which is characteristic of a thalassohaline brine. When less mobile anions or cations, involved in biological processes or oxidation-reduction processes, are discarded, we observe that the major ions Cl, Na, Ca, Mg, SO_4_, and Sr are concentrated in the brine 5.6 fold more than in overlying seawater (Table 1), which can be produced by 82% evaporation. Hence, the brine pool water can be classified as showing conservative solute behavior of the classical model “d” Na-Mg-SO_4_^22^ for evaporative brines, such as the Kebrit and Oceanographer brines^23^. Additionally, the concentration values reported for the Afifi brine pool are in agreement with a chemical evolution preceding gypsum precipitation^24^. The concentrations of Na^+^, Cl^−^ and SO_4_^2−^ point at a degree of evaporation/rehydration of evaporites between 82% and 88%^25^. The concentrations of Mg (352.9 mM) and sulfate (188.56 mM) in the Afifi brine pool are higher than those in other Red Sea brine pools (35–280 mM and 10–49 mM ranges for Mg and SO_4_^2−^, respectively^5^). The concentrations of these two ions is comparable to those in some Mediterranean Sea brines, such as the Urania brine, where the Mg and SO_4_^2−^ concentrations are 315 and 107 mM, respectively^5^. Whereas a differential dissolution of evaporites may help explain these values, the observations that sulphate concentration in the Afifi brine pool is 5.6 fold greater than that in overlaying Red Sea Water, similar to the rest of major ions, implies a limited effect of sulphate-reducing bacteria in this brine. This is in contrast to the Atlantic II Deep, which experiences a significant accumulation of metal sulphides^20^. The temperature of the brine pool is 23.3 °C, slightly higher than 22 °C in the overlying seawater column, (Fig. 2A) similarly to the majority of the other brines of the Red Sea, with the exception of brines exposed to hydrothermal events such as Discovery, Chain and Atlantis II where the temperature range from 45 to 69 °C (Table S1). The Afifi brine waters were enriched in Total Organic Carbon (TOC) compared to overlaying waters (6.7 fold increase, Table 1). The pH in the Afifi brine body drops to 5.6 from the 7.8 of the seawater overlaying the brine. The acidification of pH in the brine bodies is a trend recorded in all the Red Sea brine pools, but a pH value below 6.0 has been recorded only in Oceanographer, Kebrit and Atlantis II Deep where the pH was respectively 5.6, 5.5 and 5.0 (Table S1).

**Table 1:** Concentrations of chemical species in the brine water (370 m) and the oxic Red Sea water (320 m).

	Brine Water	Red Sea Water	Ratio BW/RSW
Al (nM)	72.91	44.44	1.6
Ca (mM)	68.64	12.33	**5.6**
Cd (nM)	0.74	1.08	0.7
Cl (mM)	3646.33	654.82	**5**.**6**
Co (nM)	<0.01	0.18	0.1
Cr (nM)	17.18	1.77	9.7
Fe (nM)	96.87	8.32	11.6
Mg (mM)	352.90	63.37	**5**.**6**
Mn (µM)	1.79	0.11	16.3
Na (mM)	3132.69	562.58	**5**.**6**
NH_4_ (nM)	248.04	347.87	0.7
Ni (nM)	<0.01	0.13	0.1
NO_2_ (µM)	0.80	0.22	3.6
NO_3_ (µM)	0.53	4.99	0.1
Pb (nM)	<0.01	0.00	
PO_4_ (µM)	37.46	1.27	29.5
SiO_2_ (µM)	98.30	14.50	6.8
Sr (µM)	604.90	108.63	**5**.**6**
Sulfate (mM)	188.57	33.86	**5**.**6**
Urea (µM)	1.57	1.27	1.2
V (nM)	<0.01	43.85	0.0
Zn (nM)	<0.01	12.53	0.0
TOC (µM)	418.1	62.7	6.7

### Prokaryote abundance and diversity in the Afifi brine

**Figure 3:**
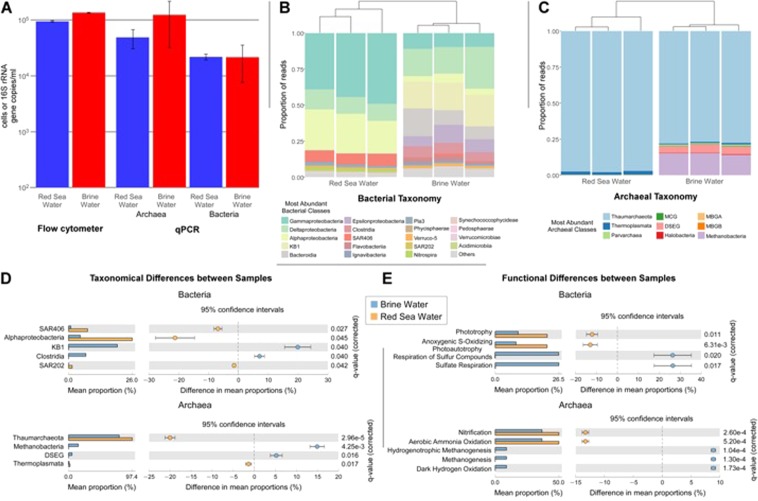
Prokaryote communities of the Afifi brine body and the oxic sea water above the brine. (**A**) Abundance of total prokaryote cells measured by flow cytometry (left histograms) and of bacterial and archaeal 16S rRNA gene copies measured by quantitative Real Time PCR (central and right histograms). (**B**) Bacterial taxonomic classes. (**C**) Archaeal taxonomic classes. (**D**) Taxonomic classes (*Bacteria* and *Archaea*) with significant differences (q ≤ 0.05) in mean proportions between the oxic deep seawater and the brine body. (**E**) Bacterial and archaeal predicted functional categories with significant differences (q ≤ 0.05) in mean proportions between the oxic deep sea water and the brine body.

Prokaryote cell abundance is significantly (p-value < 0.0001) higher in the Afifi brine than in the overlying Red Sea oxic water, with cells numbers of 1.67 × 10^5^ ± 5.59 × 10^3^ and 9.18 × 10^4^ ± 5.20 × 10^3^ cells/ml, respectively, as measured by flow cytometry (Fig. 3). This trend is similar to what was observed in other brines around the world^26,27^. Quantitative estimates of the 16S rRNA gene copy numbers of *Archaea* and *Bacteria* confirmed the flow cytometry estimates with values of 1.44 × 10^5^ ± 4.95 × 10^4^ and 7.04 × 10^4^ ± 2.08 × 10^4^ copies/ml in the brine and the overlying water, respectively, in detail, Archaea were significantly more abundant in the brine compared with the seawater (p-value < 0.01) while *Bacteria* were not (p-value = 0.959)(Fig. 3). Prokaryotes are dominated by *Archaea* both in the brine (85% of the total prokaryote 16S rRNA gene copies) and the Red Sea water (69%). The higher abundance of *Archaea* over *Bacteria* was only observed in the Atlantis II and Kebrit brines, whereas in the other Red Sea brines *Bacteria* dominate over *Archaea*^28^. These two brines are respectively characterized by high temperature (~69 °C) and a high concentration of sulfide (Table S1) which may drive the enrichment of the Archaeal community in the brines.

After quality filtering and noise-removal we obtained a total of 173,725 and 266,563 paired-end 16S rRNA genes sequences that allowed us to identify 224 and 1456 OTUs of *Archaea* and *Bacteria*, respectively. Eighty-nine OTUs of *Archaea* and 617 of *Bacteria* are shared between the brine and the overlying seawater. Both species diversity and evenness, estimated through the Shannon and the Pielou’s indices, respectively, are similar between the brine and the overlying seawater (Table 2). *Bacteria* species diversity (Shannon index) is nearly twice higher than *Archaea* whereas the two domains have similar species evenness.

**Table 2:** Shannon and Pielou’s indices of bacterial and archaeal species diversity and evenness, respectively, in the Afifi brine and the overlying oxic seawater.

	Bacteria		Archaea	
	Shannon index	Pielou’s evenness	Shannon index	Pielou’s evenness
Brine Water	3.82	0.61	2.76	0.58
	4.15	0.68	2.87	0.61
	3.90	0.62	2.83	0.60
Red Sea Water	3.53	0.57	2.66	0.65
	3.68	0.60	2.47	0.62
	4.01	0.64	2.50	0.61

**Figure 4:**
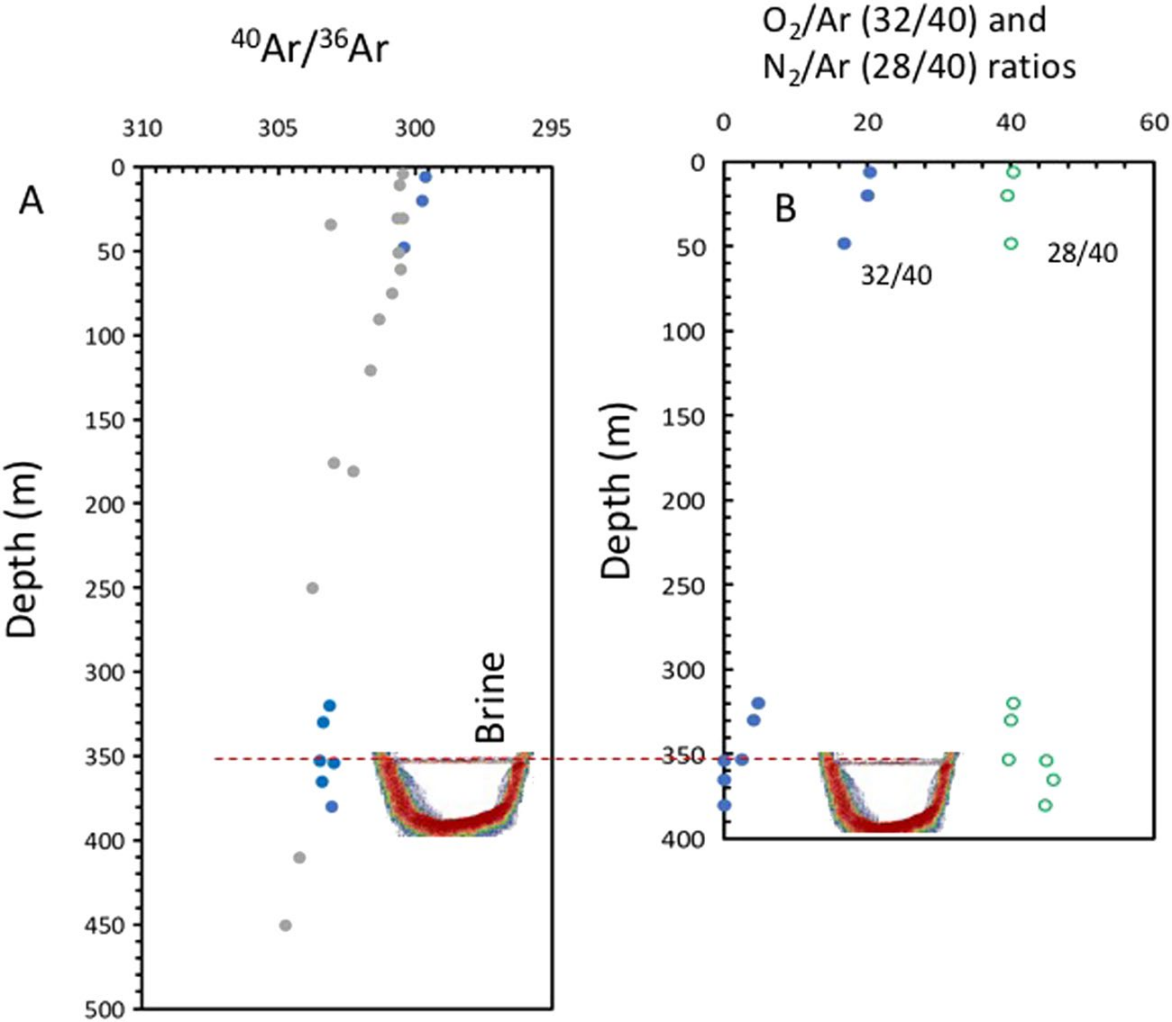
(**A**) Vertical profiles of ^40^Ar/^36^Ar ratios of a Red Sea water column at a location nearby the Afifi brine pool, with no underlying brine (grey symbols) and the Afifi brine pool, sampled on March 2, 2017 and March 3, 2017. The ^40^Ar/^36^Ar ratios in both the surface and the brine are consistent with those observed in a location where there is no brine pool. (**B**) Vertical profile of oxygen and nitrogen to argon ratios in the Afifi brine pool and overlaying Red Sea water. The icon, representing an echosounder profile of the Afifi brine pool, shows the depth where the interface between overlaying Red Sea waters and the brine pool waters is located.

The bacterial OTU taxonomic affiliation reveals that SAR406, *Alphaproteobacteria*, and SAR202 are significantly higher in the Red Sea water while KB1 and clostridia classes are specific of the brine (Fig. 3). Hierarchical clustering of all samples based on the normalized abundance for each bacterial class indicates a clear separation between the communities associated to the anaerobic and highly saline brine water and the overlying oxic seawater. The dominant classes in the Red Sea water are *Gammaproteobacteria*, *Deltaproteobacteria*, *Alphaproteobacteria*, and SAR406 accounting for more than 90% of the bacterial sequences. *Deltaproteobacteria*, *KB1*, *Bacteroidia*, *Gammaproteobacteria*, *Epsilonproteobacteria* and Clostridia that compose 81% of the bacterial community, dominate in the brine (Fig. 4). The relative abundance of some bacterial taxa such as KB1 and clostridia significantly (p < 0.05) shifts in the brine where the conditions turn anaerobic, with proportions, respect to the overlying seawater, increasing from 0% to 20% and 8%, respectively. Furthermore, the presence of KB1 bacteria only in the brine body can be explained by the metabolic adaptation of these bacteria to the high salinity encountered in this environment^29^. *Alphaproteobacteria*, SAR406 and SAR202 significantly decreases in the brine of 19%, 7%, and 1.5%, respectively (Fig. 3).

The archaeal OTU taxonomic affiliation revealed that *Thaumarchaeota* and *Thermoplasmata* are significantly higher in the overlying Red Sea water than in the brine, while *Methanobacteria* and DSEG (Deep Sea Euryarcheota Group) classes were typical of the brine (Fig. 3). Similarly to bacterial classes, the two environments are clearly separated based on the normalized abundance for each archaeal classes. The *Thaumarchaeota* class composes nearly all the archaeal community in the oxic sea water (97.4%). In the brine, the archaeal sequences are dominated by the *Thaumarchaeota* and *Methanobacteria* classes that, together, accounts for more than 94% of archaeal sequences. The relative abundance of some archaeal taxa also shifts between the Red Sea water and the brine. The *Methanobacteria* and DSEG classes are absent in the Red Sea water, whereas they compose 11% and 4% of the community in the brine.

With the notable exception of the MSBL1 archaeal group absent in Afifi, most of the taxa abundant in the brine body were also found in other brines of the Red Sea, including the KB1 clades^28,30^.

Functional assignments deduced from the bacterial and archaeal taxonomy indicates that methanogenesis and respiration of sulfur compounds are significantly higher in the brine than in the overlying seawater (Fig. 3). Using the FRAPOTAX software^31,32^, we were able to functionally assign 25% and 26% of the archaeal and bacterial OTUs accounting for 75% and 32% of the community abundance, respectively. This relatively low abundance of functional assignment is probably due to relatively unstudied brines environments with many new microorganisms belonging the so-called microbial dark matter. In the seawater overlying the brine, most of *Thaumarchaeota* members were associated to nitrification whereas bacteria of the *Gammaproteobacteria* class were predicted to be phototrophs. The presence of phototrophs bacteria in the seawater and in the brine waters communities can be explained by the shallow depth of the Afifi basin and the fact that they belong to the Ectothiorhodospiraceae family which are purple sulfur bacteria capable of photosynthesis^33^. In the brine, members of the *Deltaproteobacteria* class resulted associated to sulfur or sulfate respiration, while the *Methanobacteria* class to methanogenesis. The increasing in abundance of Deltaproteobacteria associate to sulphur and sulphate respiration and Methanobacteria involved in the methanogenesis, confirmed the variability of metabolism adopted by microorganisms to thrive in the harsh brine environment and how these microbes are adapted to the anoxic conditions of the brine waters. The respiration of sulphur compounds has been widely detected in different brines where high sulfate reduction rate has been observed, both in sulphidic brine like Kebrit, and in non sulfidic brine such as Atlantis II, Discovery, Nereus and Erba, where it was also hypothesized the presence of specific sulfate reducing bacteria adapted to the condition of the brines^28^.

### Stable isotope composition of water, major gases and nitrate

δ^36^Ar and δ^38^Ar in the dissolved gases have values close to atmospheric equilibrium in Red Sea water, with ^40^Ar/^36^Ar ratios around 299.5 (atmospheric equilibrium), slightly more enriched in ^40^Ar that the atmospheric air (296.16^34^). These values are higher in the Afifi Brine Pool, ranging between 303.06 and 303.50 (Tables 3 and 4, Fig. 4A). Winckler *et al*.^35^ found excess in ^40^Ar (ratios ^40^Ar/^36^Ar as high as 305) in other brines of the Red Sea (Atlantis II and the Discovery) that these authors interpret as mantle-derived Argon, as the ratio ^40^Ar/^36^Ar of the mantle is around 42,450^36^. Hence, isotopic balance calculations show that a mantle gaseous contribution of about 0.01% would suffice to justify the values found in the Afifi brine pool. However, for Afifi pool we found a similar trend in values in ^40^Ar/^36^Ar ratios at similar depths in a nearby location where the brine pool is not present (Fig. 4A). Therefore, the ^40^Ar/^36^Ar ratios in the Afifi brine pool are more parsimoniously explained as the result of enrichment in ^40^Ar related with isotopic fractionation due to diffusion processes in the ocean, an observation which does not require invoking a small contribution of radiogenic argon (cortical or mantle gases) related to the submarine volcanism of the Red Sea.

**Table 3:** Isotopic composition of dissolved gases, TIC and nitrate in the Afifi brine pool waters and overlaying Red Sea waters.

Depth	δD	δ^18^O	δ^13^C (TIC)	[TIC]	δ^18^O (NO_3_^−^)	δ^15^N (NO_3_^−^)	[NO_3_^−^]
(m)	(SMOW)	(SMOW)	(V-PDB)	µmol/Kg	(SMOW)	(AIR)	µmol/Kg
6	11.2	1.17	0.26	2173	14.4	5.9	0.7
20	10.0	0.86	0.29	2135	12.9	5.6	0.7
48	12.3	2.07	−0.05	2201	9.5	3.9	2.6
320	8.2	1.46	−0.60	2296	4.7	2.7	12.4
330	10.5	1.64	−0.66	2313	4.9	2.9	11.5
353	15.1	2.35	−5.29	2782	12.6	6.9	0.8
354	22.9	4.11	−9.19	3328	18.4	9.8	0.8
365	25.6	4.26	−9.01	3345	16.8	13.5	0.7
380	25.7	4.55	−9.44	3363	18.3	10.1	0.8

Samples collected at depths 354–380 correspond to the Afifi pool waters, samples from 353 correspond to the interface with the overlaying Red Sea water, and samples shallower than 353 correspond to Red Sea water unaffected by the Afifi pool brines.

**Table 4:** Isotopic composition and ratios of O_2_, N_2_ and Ar in the Afifi brine pool waters and overlaying Red Sea waters.

Depth	δ^15^N	δ^17^O	δ^18^O	δ^36^Ar	δ^38^Ar	^40^Ar/^36^Ar	^38^Ar/^36^Ar	^40^Ar/^38^Ar	O_2_/N_2_	O_2_/Ar	N_2_/Ar	(O_2_ + N_2_ + Ar) = 100%		
(m)	(AIR)	(SMOW)	(SMOW)	(AIR)	(AIR)				32/28	32/40	28/40	% O2	% N2	% Ar
6	0.28	6.30	21.71	−11.53	−38.75	299.6	0.1825	1642.1	0.5109	20.3292	40.4333	32.92	65.47	1.62
20	0.61	6.19	21.60	−11.93	−42.60	299.7	0.1818	1648.7	0.5143	20.0034	39.5538	33.03	65.32	1.65
48	0.42	7.67	22.82	−14.17	−42.05	300.4	0.1823	1647.7	0.4239	16.7977	40.1393	28.99	69.28	1.73
320	1.14	14.48	24.84	−22.97	−42.92	303.1	0.1838	1649.2	0.1178	4.8351	40.5040	10.43	87.41	2.16
330	1.10	13.88	23.74	−23.77	−47.67	303.4	0.1830	1657.4	0.1023	4.1144	40.0358	9.11	88.67	2.21
353	3.00	25.97	23.36	−24.17	−35.47	303.5	0.1855	1636.5	0.0642	2.5789	39.7353	5.95	91.74	2.31
354	6.66	—	—	−22.50	−25.29	303.0	0.1871	1619.4	0.0009	0.0138	45.0318	0.03	97.80	2.17
365	6.88	—	—	−23.81	−26.13	303.4	0.1872	1620.8	0.0000	0.0057	46.0025	0.01	97.86	2.13
380	6.80	—	—	−22.76	−26.25	303.1	0.1870	1621.0	0.0000	0.0023	44.7768	0.01	97.81	2.18

Samples collected at depths 354–380 correspond to the Afifi pool waters, samples from 353 correspond to the interface with the overlaying Red Sea water, and samples shallower than 353 correspond to Red Sea water unaffected by the Afifi pool brines.

**Figure 5:**
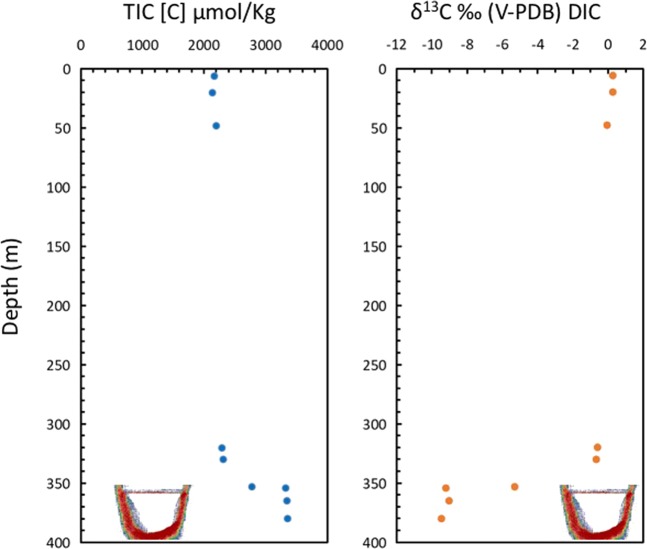
Concentration and carbon isotopic composition of Total Inorganic Carbon (TIC) in the Afifi brine pool waters and the overlaying Red Sea water. The icon, representing an echosounder profile of the Afifi brine pool, shows the depth where the interface between overlaying Red Sea waters and the brine pool waters is located.

The Afifi brine pool water is enriched in total inorganic carbon (TIC), which reaches values of 3300 µmol L^−1^ compared to typical values of around 2300 µmol L^−1^ at similar depths (Table 3, Fig. 5). isotopic values of TIC change drastically in the brine pool. This enrichment in TIC by about 1000 µmol L^−1^ is accompanied by very negative δ^13^C isotopic values of TIC, of around −11‰, compared to the typical values of around 0‰ (V-PDB) in the water column (Fig. 5). The negative TIC isotopic values requires a very negative source of TIC, compatible with that expected to be produced by respiration of oil oxidation or methane oxidation. The CO_2_ released would have values close to −30‰ (V-PDB), compatible with both oil or methane of thermogenic origin^37–41^. Anaerobic oxidation of methane (AOM) would produce oxygen produced through denitrification and nitrite or sulfate reduction, which be used, in turn, to oxidize methane, producing a CO_2_ with very negative δ^13^C values in the brine, as supported by the increase of *Epsilonproteobacteria* and *Deltaproteobacteria* in the brine. Consequently, AOM and associate denitrification would explain both isotopic tendencies, TIC towards very negative δ^13^C values and high values of d^15^N in nitrates and N_2_ (Tables 3 and 4).

**Figure 6:**
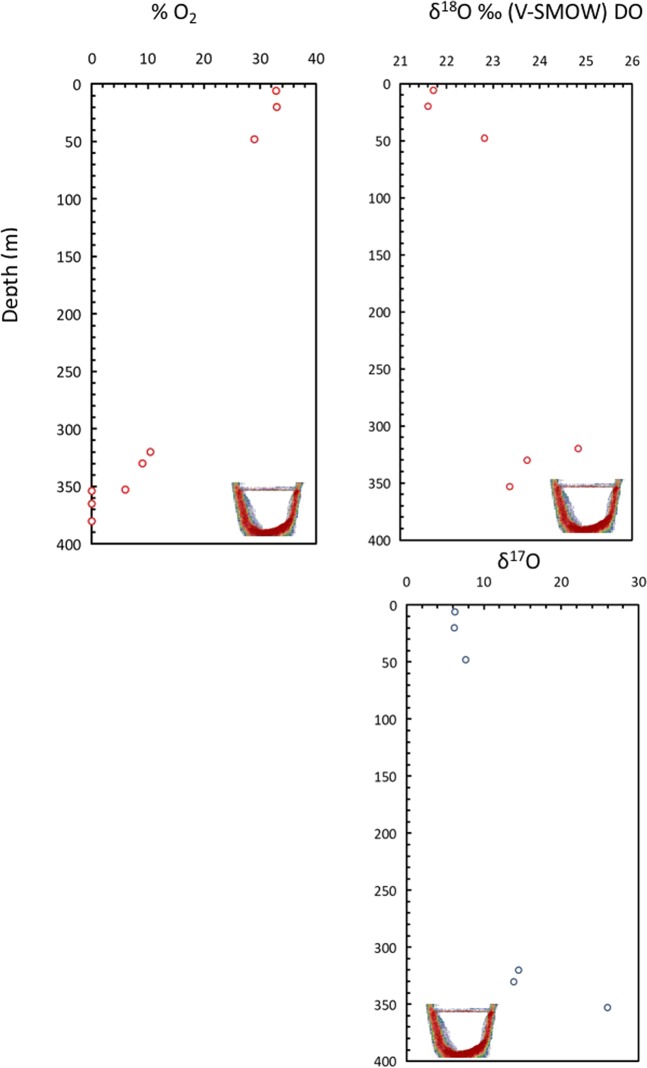
Vertical profiles of (**A**) Percentage of oxygen, relative to the sum of all major dissolved gases (N_2_ + O_2_ + Ar), excluding CO_2_. Isotope composition of δ^18^O (**B**) and δ^17^O (**C**) of the dissolved oxygen in the Afifi brine pool waters and the overlaying Red Sea water. The icon, representing an echosounder profile of the Afifi brine pool, shows the depth where the interface between overlaying Red Sea waters and the brine pool waters is located. Note that that no isotopic oxygen data is reported inside the brine, since the concentration was too low to resolve this.

The Afifi brine pool is anoxic with oxygen concentrations ranging from below detection limit in the brine pool interior to 5.97% at the brine-Red Sea water interface (Table 3, Fig. 6). The water at the brine-Red Sea water interface has relatively high δ^18^O values (23.4 to 24.8‰ V-SMOW, Tables 3 and 4, Fig. 6), consistent with isotopic fractionation derived from a predominance of respiration and the preferential use of the light ^16^O vs ^18^O and ^17^O^42–44^. The δ^17^O values are also anomalous with values as high as +25.97‰ (V-SMOW) related with respiration^43^. This isotopic balance explains the increase in ^18^O and ^17^O in the dissolved oxygen in the waters at the interface above the brine pool (Table 3, Fig. 6), while the low contents of dissolved oxygen involve relative increases in N_2_ and Ar concentrations (Fig. 4B). The decrease of dissolved oxygen concentration in the brine waters explains why in this layer there is an enrichment of microbes with anaerobic metabolisms that are not present in the overlaying seawater.

**Figure 7:**
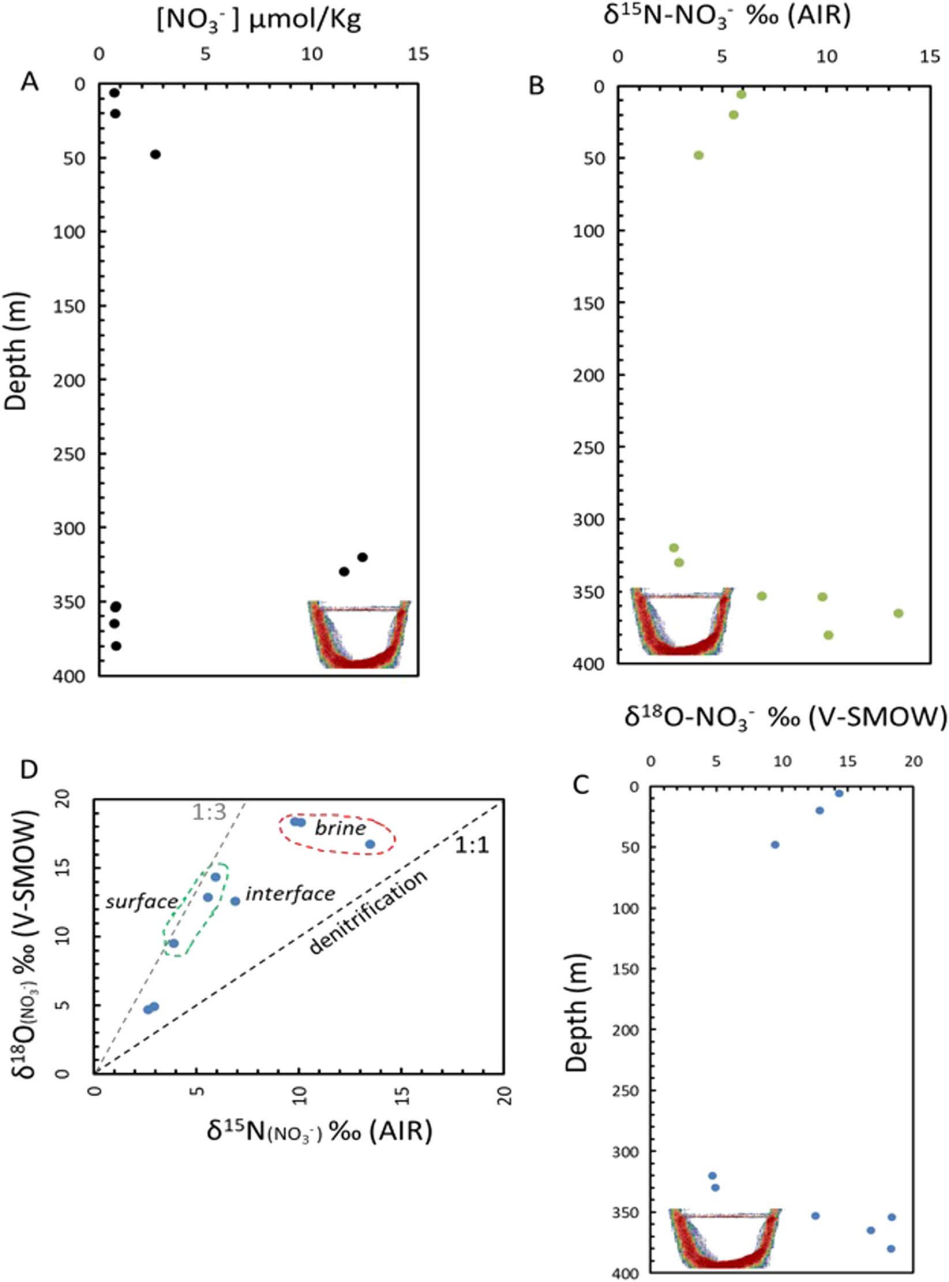
Vertical profiles of (**A**) nitrate concentration, (**B**) the isotopic composition o nitrogen in nitrate, (**C**) the isotopic composition of oxygen in nitrate in the Afifi brine pool waters and the overlaying Red Sea water, and (**D**) the relationship between the isotopic composition of oxygen and nitrogen in nitrate in the water column of the Afifi brine pool. The icon, representing an echosounder profile of the Afifi brine pool, shows the depth where the interface between overlaying Red Sea waters and the brine pool waters is located.

**Figure 8:**
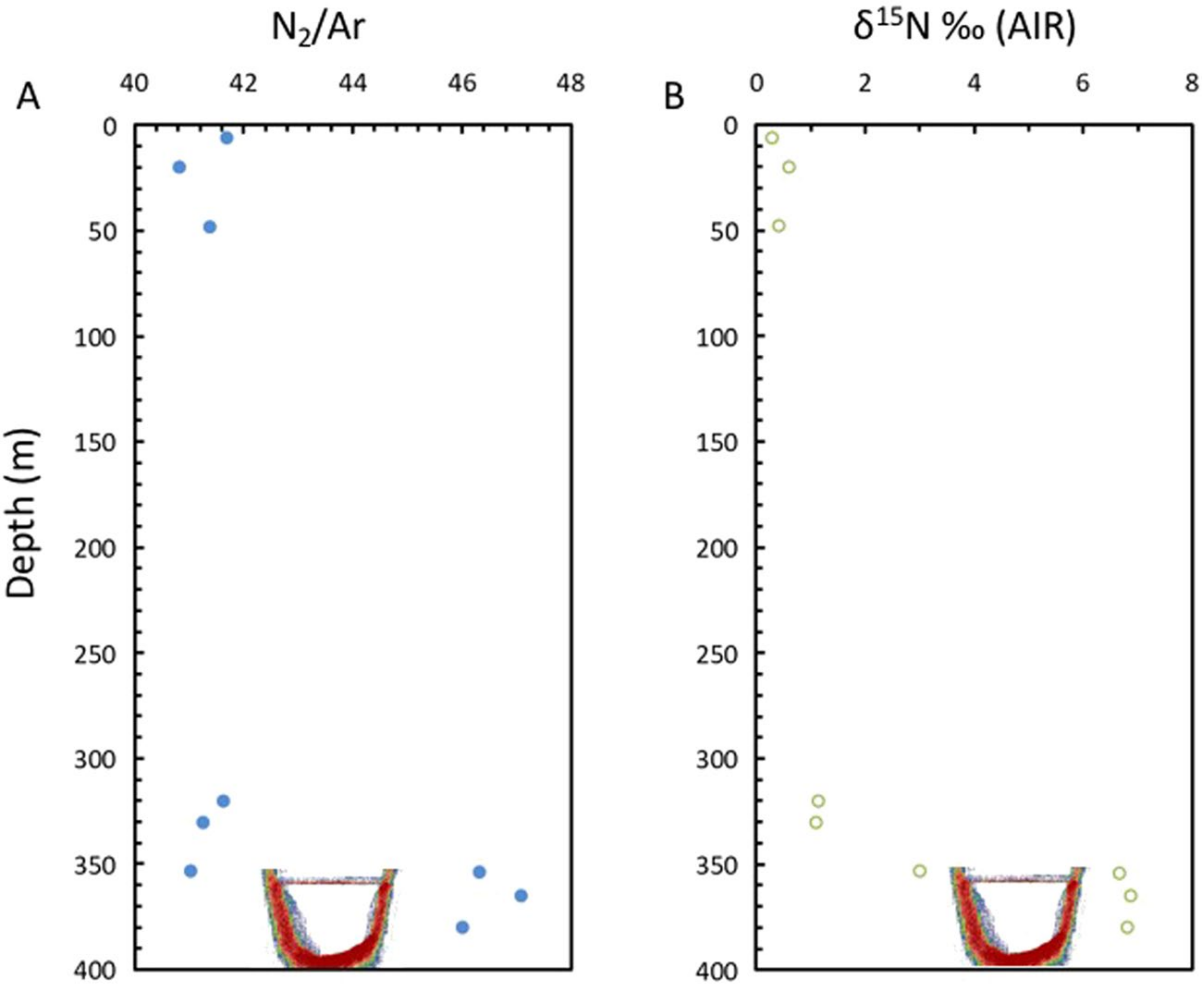
Vertical profiles of (**A**) nitrogen to argon ratios, and (**B**) the isotopic composition of nitrogen (N_2_) in the gasses dissolved in the waters of the Afifi brine pool. The icon, representing an echosounder profile of the Afifi brine pool, shows the depth where the interface between overlaying Red Sea waters and the brine pool waters is located.

A remarkable enrichment in ^15^N in the dissolved nitrogen of the brine pool waters was observed, reaching δ^15^N values of +6.88‰ (AIR, Table 3, Fig. 7), quite extreme for the marine environment^45,46^. This observation is in contrast with the normal values of the water column unaffected by brines at comparable depths, ranging between δ^15^N values of +0.28 and +1.14‰ (AIR, Table 3, Fig. 7). The ratio O_2_/Ar ranged between 0.0057 and 0.0138 in the brine and 2.6 and 4.8 in waters just above the brine pool surface interface, compared to values about 16.8 to 3.03 in the water column (Fig. 4B). The anoxic environment of the Afifi brine pool leads to denitrification processes and reduction of nitrates/nitrites to NO, N_2_O and N_2_. The trend toward high values of δ^15^N and δ^18^O in nitrates is in agreement with denitrification processes in the brine (and brine interface) and assimilation in surface waters (Figs. 7 and 8). The overlaying Red Sea water with higher nitrate concentration, delivered with advected Arabian Sea waters, show values around +3‰ for δ^15^N and +5‰ for δ^18^O, typical of the deep ocean^45,46^ (Figs. 7 and 8), while the values in the brine waters indicate a reduction of nitrate to nitrite and denitrification processes of nitrites to NO, N_2_O and N_2_ (Figs. 7 and 8). Additionally, the presence of NH_3_ and NO_2_^−^ enable the processes of anammox and the generation of N_2_, as supported by the presence of *Candidatus Scalindua* and other anammox bacteria in brines of the Red Sea^5,30^. Denitrification/anammox processes could release N_2_ highly enriched in ^15^N^47^, which would explain these isotopic changes from the background of the atmospheric dissolved N_2_ toward high δ^15^N values in the brine. This is also in agreement with the increase in N_2_ observed in brine waters (excess of N_2_), as indicated by N_2_/Ar ratios of 46 in brine waters compared to 41 at similar depths in waters unaffected by brines (Figs. 4B and 8). N_2_ contributions from mantle gas would be inconsistent with our observations, as δ^15^N values in mantle gas are usually slightly negative^48^. Degradation of organic matter in the brine waters and the sediment undelaying the brine pool and release of N_2_ relatively enriched in ^15^N would also contribute to the observed δ^15^N values^49^. However, in the brine layer of the Afifi Brine, members of the Planctomycetes phylum that are anammox bacteria were only found in very low abundance (1.2%) which suggest that the δ^15^N value are more probably due to denitrification processes. However, we cannot exclude that a relatively high proportion of these metabolisms occur in the brine seawater interface that constitutes a ﻿functional hotspot of the brines^5^.

**Figure 9:**
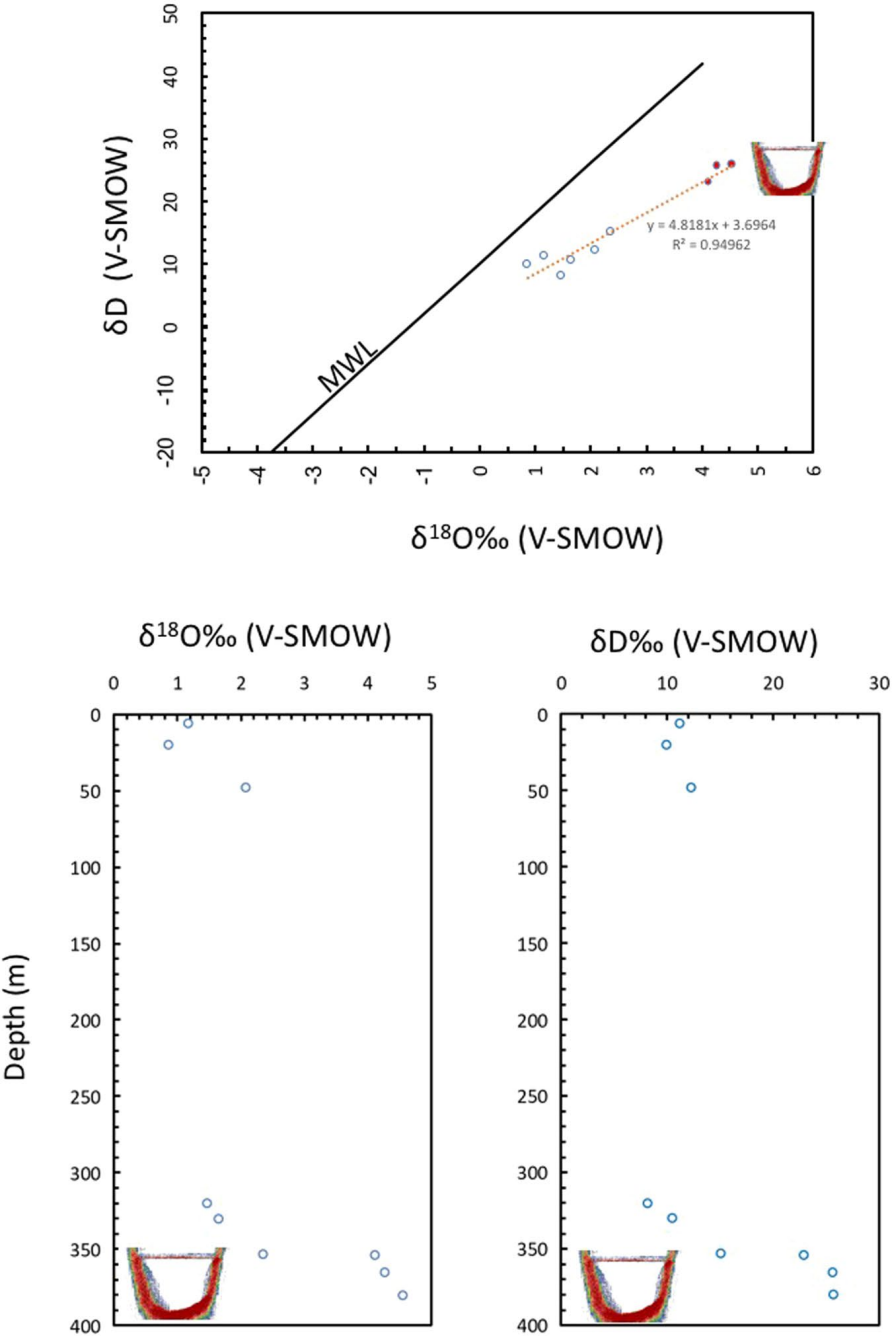
The relationship between oxygen and deuterium isotopes in waters of the Afifi brine pool (red symbols) and overlaying Red Sea waters (open symbols), and the corresponding vertical profiles of these isotopes in the waters of the Afifi brine pool. The icon, representing an echosounder profile of the Afifi brine pool, shows the depth where the interface between overlaying Red Sea waters and the brine pool waters is located. The solid line shows the relationship expected under evaporation (MWL) and the dotted line shows the fitted regression line, corresponding to the equation shown.

The δ^18^O isotopic value of the water in the pool is more positive that in the water column (Fig. 6). The increasing in δ^18^O isotopic values in some Red Sea brine waters has been explained in the past invoking hydrothermal processes^14^. However, the Afifi brine pool has waters with high isotopic values in both oxygen and hydrogen (Fig. 9). A hydrothermal system would justify the high values of oxygen, but not those of hydrogen^50^. In fact, subaerial hydrothermal water in the same general basin of the Red Sea-crossing basalt and evaporitic sediment, has high δ^18^O values of around +10‰ (V-SMOW) but little change in δD^51,52^. The Afifi brine pool and column waters showed a linear relationship between δ^18^O and δD values that is compatible with evaporation processes (δD = 4.82 δ^18^O + 3.67; R^2^ = 0.95, Fig. 9). The slope of this relationship is similar to that of evaporation experiments reported by Craig *et al*.^53^, and similar to the evaporation line for the Red Sea continental area and Red Sea brines reported by Gonfiantini *et al*.^51^. Likewise, the slope of this relationship represents intermediate values between low temperatures and high temperatures of crater lakes^54,55^. The brine pool waters, therefore, have an isotopic footprint of the typical enrichment in ^18^O and D expected from evaporative processes^56^, possibly derived from seepage of fossil brines from evaporitic sediments, to yield brine pool waters 5.6 times saltier than the overlying Red Sea waters.

The geological setting of the Afifi pool is between Plio-Pleistocene reef platforms underlain by Miocene layered evaporites, including salt^9^. Salt tectonic processes formed diapirs that flowed on the seafloor^52^, and the dissolution of salt may have formed the brines that accumulated in the Afifi bathymetric depression. We suggest that the water in the Afifi brine pool was derived from seawater trapped in the pores of the underlying evaporitic sediments, which would have undergone important isotopic fractionation due to evaporation (intrinsic to the genesis of evaporites)^57^. A mixture of normal waters of the Red Sea and these waters trapped in the evaporites (formation or diagenetic water), could give rise to these isotopic and salinity values. In addition to the water present in the pores of the sediments, gypsum contains about 21% of structural water with values usually enriched in ^18^O and ^2^H^58^, a transformation of gypsum (CaSO_4_.2H_2_O) to anhydrite (CaSO_4_) causes the release of water can be in agreement with the apparent line of evaporation of this study. This last hypothesis explains both the chemical and isotopic composition aspects of the waters of Afifi brine pools. Other diagenetic waters are related to alteration of volcanic rock to clay minerals, which generates water impoverished in ^18^O, but enriched in D, because the mineral phase is enriched in ^18^O and impoverished in D^50,59–62^. This process may be common since bottom sediments are rich in montmorillonite^20^. Conversely, diagenetic water in equilibrium with inherited clay mineral have negative δD values^63^. The Salam Field in the Western Desert of Egypt has, at present, slightly negative isotopic values of δ^18^O = −0.59‰ and δD = −13.5‰^64^. A large dispersion of values was found by^65^, who concluded that the water formed often has positive in oxygen but negative in hydrogen isotopic δ values. Brine waters dominated by hydrothermal influences would have enriched δ^18^O values, but no difference in δD^13^. Accordingly, our results indicate that the Afifi brine pool water is not hydrothermal in origin.

A long distance migration of continental brines to the Red Sea deeps has been proposed by other authors^66^, which we consider highly unlikely. However, the basin containing the relatively shallow Afifi brine pool (Fig. 1) could have been semi-isolated from the Red Sea during a possible closure of the Red Sea during the last glaciation, when sea level was 120 m lower than present^67^. In this scenario, the Afifi brine pool could be largely the result of a glacial lowstand evaporative event, accounting for the agreement between the isotopic composition of its waters and a theoretical evaporation line of Gofiantini *et al*.^51^ and the concentration of cations and anions associate to degree of evaporation of seawater by 82–88%^25^. For example, seawater evaporation in pools of a natural salt factory (Cabo de Gata, SE Spain) give similar isotopic values to those found in the Afifi brine pool^58^. However, this hypothesis is inconsistent with the ionic composition of Afifi pool waters, which does not provide evidence of precipitation of sulfates or other minerals. Consequently, we believe that the most likely hypothesis explaining the origin of the Afifi brine pool brines are submarine seeps of pore (formation) waters derived from seawater after diagenetic reactions, and which became concentrated brines from dissolution of the underlying evaporitic sediments.

The Afifi brine pool reported and named here is the shallowest and southern-most brine pool yet known in the Red Sea, and offers, because of its accessibility, a convenient test bed to study microbial and geochemical processes in brine pool waters. Most importantly, the unique isotopic composition of the Afifi brine waters also suggest an origin different from that of other Red Sea brine pools, dominated by interactions with evaporites without any contribution of hydrothermal fluids, which is different from that of the hot brine pools in the Red Sea. This finding adds to the diversity of environments and processes yielding Red Sea brine pools and calls for a revision of the theories explaining their origins.

## Methods

### Acoustic survey and water sampling

Acoustic surveys leading to the discovery and characterization of the Afifi brine were conducted in three research cruises on board R/V Thuwal, 20–27 September 2016, 31 March to 7 April 2017 and 23 November to 7 December 2018. During the first cruise the brine was detected by a Simrad EK60 hull-mounted echosounder operating at 38 kHz, with a 7 degree vertical acoustic beam. A grid acoustic survey was conducted to map the brine, and based on the echosounders bottom and brine surface detections, a 3D model of the brine was created in Matlab. From this model brine area and volume were calculated.

To make a high resolution bathymetry map (Fig. 1), a newly installed multibeam system was used in a grid survey in the last cruise, a Kongsberg EM 710 Mk2, with 2 degree beams and frequencies from 40–100 kHz and a swath coverage sector up to 140 degrees. The survey was done with a 200% swath overlap and with focused beams, maximizing resolution and decreasing post processing of data. All post processing used the QPS Qimera and Fledermaus software with final map being generated in ArcGis 10.3.1.

Sampling of the Afifi Brine (17.56 N, 41.46 E) has been carried out with the R/V Thuwal on April 2017 using a rosette system equipped with 23 Niskin bottles and an Idronaut® CTD for measuring temperature, salinity and pH. The salinity and pH were further controlled when the samples were onboard using a portable multi-parameter detector (YSI). We sampled triplicates Niskin bottles at each depth, 30 m above the seawater/brine interface (depth: 320 m) and 20 m below (depth: 370 m). Once on the deck, we filtered five liters of the recovered seawater or brine on 0.2 μm sterile polyethersulfone Sterivex^TM^ (Millipore Corporation) by using peristaltic pumps (Millipore) for the subsequent DNA extraction. The filters were stored in tubes filled up by lysis buffer (EDTA 40 mM pH 8.0, Tris-HCl 50 mM pH 8.0, sucrose 0.75 M) in liquid nitrogen. Triplicate seawater and brine samples of 1.62 mL per each depth were fixed with 180 μL of paraformaldehyde and glutaraldehyde fixing solution for a total volume of 1.8 mL per sample. The samples were mixed and incubated in the dark at room temperature for ten minutes and stored at −20 °C until further analysis. We prepared the fixing solution at a final concentration of 10% phosphate buffer, 10% paraformaldehyde and 0.5% paraformaldehyde and we stored it at −80 °C. The pH was adjusted at 7.4 adding NaOH pellets. 15 ml of filtered water was stored in duplicates for further chemical analysis. The major ion composition of the brine water and the oxic Red sea water was determined using the commercial service provided by GEOMAR Helmholtz Centre for Ocean Research, Kiel (Germany).

### Flow cytometry

The samples were analyzed with an Attune NxT acoustic focusing cytometer (Thermo Fischer Scientific). The cells were stained with Sybr Green I nucleic acid gel stain (Life Technologies) at 10X final concentration. After staining, we incubated the samples in the dark for 20 minutes and then transferred in a 96-well-plate (Axigen, Corning Incorporated) to read them with the instrument’s autosampler. Sterile deionized water was used to clean the instrument after each measurement. The 96-well-plate was shacked before each measure for one cycle, and after each well, the flow cytometer was washed for three cycles. We set the voltages as follow on the different channels: Front Scatter (FSC) 350 mV, Side Scatter 350 mV, BL1 (Sybr Green) 430 mV with the following respective thresholds FSC 100, SSC 400, BL1 500. For each sample, we analyzed a total volume of 50 μL with a speed of 12.5 μL per min. We started to record the events after 30 secs.

Two negative samples for the Red Sea water and the brine water were run to gate possible false events: one non-filtered and non-stained sample and one filtered (0.2 μm pore size) and Sybr stained sample. For the exact gating strategy, please refer to Figs. S1 and S2. The final cell counts were multiplied by 1.1 to account for the dilution due to cell fixation.

### DNA extraction

The phenol-chloroform extraction protocol was used to extract total DNA from the different filters. First, 20 mg/mL of lysozyme were added to the filter and then incubated at 37 °C in a water bath for 30 minutes. After this step, 20 mg/mL of proteinase K and 20% of the final solution volume of SDS were added and then incubated at 55 °C for 2 hours in a water bath. The supernatant was transferred to a new sterile 15 mL Falcon tube and 1 volume of phenol:chloroform:isoamyl alcohol (25:24:1, pH 7.7–8.3) was added. The sample was shacked for 30 seconds and centrifuged at 8,000 g for 10 minutes. The aqueous phase was then transferred to a new sterile 15 mL tube. The phenol:chloroform:isoamyl alcohol step was repeated a second time. Then, a volume of chloroform:isoamyl alcohol (24:1) was added followed by another shaking and centrifugation step for 10 minutes. The aqueous phase was transferred in a new Falcon, and 2 volumes of ice-cold 100% ethanol with 1/10 volume of sodium acetate (3.0 M pH 5.3) were added. Following an overnight incubation at −20 °C, the sample was centrifuged at 4 °C to precipitate the DNA and the supernatant was discarded. The precipitated DNA was washed in two further steps with ice-cold 80% ethanol alternated to 4 °C centrifugation step before being resuspended in Milli-Q water and stored at 4 °C or at −20 °C. The extracted DNA of all samples was quantified by Qubit® 3.0 Fluorometer using the Qubit® dsDNA BR assay kits (Thermo-Fisher Scientific), and the quality was assessed by electrophoresis on 1% agarose gels.

### Quantitative PCR

Absolute abundances of bacterial and archaeal 16S rRNA gene copies were determined by quantitative PCR by using the primer-sets Eub338/Eub518 and Arc931F/Arc1100R, respectively^68,69^ (Fierer *et al*. 2005, Einen *et al*. 2008). To generate the reference DNA calibration standard curve for bacteria and archaea amplicon fragments of ±180 bp for *Bacteria* and ±170 bp for *Archaea* were cloned in a plasmid of *E*. *coli*. The plasmids were extracted with the Pure Yield Plasmid Miniprep (Promega) and quantified using the Qubit dsDNA HS Assay Kit (Thermo-Fisher Scientific). Series of standards were prepared through ten-fold serial dilutions and stored at −20 °C. Quantitative PCR reactions were carried out on a Rotor-Gene Q thermocycler (Qiagen). We prepared the reaction mixes with the GoTaq^®^ qPCR Sybr Green Master Mix (Promega). The final volume was 15 μl, containing 1X GoTaq^®^ Master Mix, 100 nM of each primer and 1.5 μl of template DNA. Quantitative PCR conditions were the following: 95 °C for 2 minutes, 40 cycles at 95 °C for 15 seconds, 53 °C (*Bacteria*) or 64 °C (*Archaea*) for 20 seconds and 60 °C for 20 seconds; at the end of the run, melting curves of the PCR products were obtained through 91 cycles from 50 °C to 95 °C with increase of 0.5 °C/cycle every 5 seconds. We constructed standard curves with a series of dilutions ranging from 50 to 5 × 10^7^ copies/μl. We ran all the standards and samples in triplicates. R^2^ values and amplification efficiency for *Bacteria* were 0.99835 and 103% respectively, while for *Archaea* 0.99555 and 109% respectively. Conversion from 16S rRNA gene copy numbers to bacterial cell number considered the average 16S rRNA gene copy number (GCN) of each sample obtained using Copyrighter^70^, using the prokaryotes profiles of the 16S rRNA Illumina MiSeq libraries.

### 16S rRNA high-throughput gene analysis

The 16S rRNA libraries on all the extracted DNA were generated using the Illumina® Nextera XT Sample Prep Kit, following the Illumina® 16S metagenomic sequencing library preparation protocol for sequencing the 16S rRNA genes V3 and V4 variable regions of bacteria and V4 region of archaea. The primer pairs Bac341F (CCTACGGGNGGCWGCAG) and Bac785R (GGATTAGATACCCVHGTAGTC) and Arch519F (CAGYMGCCRCGGKAAHACC) and Arch806R (GGACTACNSGGGTMTCTAAT) were used for generating the bacterial^71^ and archaeal^72^ libraries, respectively. Noise filtering and sequence clustering into operational taxonomic units (OTUs) were performed using the UClust algorithm^73^ in QIIME v1.9^74^, as described by Fodelianakis *et al*.^75^. Taxonomic assignment for each OTU was done using the Greengenes database (v13_8^76^). Statistical analyses were performed using the R packages phyloseq and vegan^77,78^. Singletons OTUs were discarded and sequence counts were log(x + 1) transformed. Species richness and evenness were estimated with the Shannon and the Pielou’s indices, respectively. Hierarchical clustering using the Euclidean distance metric and average linkage was used to cluster all samples based on the normalized abundance for each domain. Functional annotations of prokaryotic clades was done using the FAPROTAX software which uses current literature on cultured strains to assess metabolic functions^31,32^. The taxonomy of the different OTUs was thus used to determine the potential function of the microbiome. Significant differences between taxa or functions were calculated using White’s non-parametric t-test at a 95% confidence interval with a Benjamini-Hochberg FDR correction using the STAMP software^79^.

### Stable isotopes

Isotopic measurements were carried out at the Stable Isotope Laboratory of Andalusian Earth Sciences Institute (CSIC). For the analysis of δD an aliquot of water (0.7 µl) was injected onto a ceramic column containing a glassy carbon tube at 1450 °C to produce H_2_ and CO gases^80^. A high-temperature reactor (TC/EA) coupled on-line via a ConFlo III interface to a Delta XP isotope ratio mass spectrometer (Thermo-Finnigan, Bremen). These gases were separated by chromatography using a helium carrier gas stream. To avoid memory effects, each sample was analyzed 10 times with the dis first 5 analyses discarded the last five averaged to yield the estimate used. δ^18^O in water was analyzed using the CO_2_ – H_2_O equilibration method^81,82^. Commercial CO_2_ and H_2_ bottles and 5 different waters, previously calibrated vs. V-SMOW, SLAP and GIPS, were used as internal standards for the oxygen and hydrogen isotopic analyses. The precision was calculated higher than ±0.1‰ for the oxygen and ±1‰ for the hydrogen, while the standard for reporting oxygen and hydrogen is V-SMOW (Vienna Standard Mean Ocean Water).

For the TIC (Total Inorganic Carbon) an aliquot of sample was injected into 12-ml vials pre-filled with helium and 5 drops of 65% phosphoric acid and shaken in a Vortex agitator for 30 seconds. The vials were left at room temperature for between 15 and 36 hours to obtain a state of equilibrium^83^. The CO_2_ was separated from other residual gases by chromatography using a helium carrier gas in a Gas Bench (ThermoFinnigan, Bremen, Germany) system interfaced with a mass spectrometer Delta XP isotope ratio mass spectrometer (Thermo-Finnigan, Bremen). Samples for total organic carbon (TOC) were acidified with H_3_PO_4_ until pH 1–2, and kept in the dark at 4 °C until analysis by high temperature catalytic oxidation at the laboratory using a Shimadzu TOC analyzer. Glass material used was previously acid cleaned and burned (450 °C, 4.5 h), and reference material of deep sea carbon (42–45 μmol C L^−1^) and low carbon water (1–2 μmol C L^−1^), provided by D. A. Hansell and Wenhao Chen (Univ. of Miami), were used to assess the accuracy of TOC estimates.

Dissolved gases: The ratios O_2_/Ar, N_2_/Ar, CO_2_/Ar, O_2_/N_2_ and isotopic composition of ^15^N/^14^N, ^18^O/^16^O, ^17^O/^16^O, ^36^Ar/^40^Ar, ^38^Ar/^40^Ar were analyzed in a system built specifically for analysis of atmospheric gases by continuous flow. The system employed a Delta V plus (ThermoFinnigan, Bremen, Germany) mass spectrometer configured with 11 faraday cups to simultaneously analyze N_2_, O_2_, CO_2_ and Ar. This allowed the maximum precision in the mixture of atmospheric gases. The precision was calculated for 4 bottles of internal standards vs air and was better than ±0.03‰ for δ^15^N, ±0.05 for δ^17^O, ±0.02 for δ^18^O, ±0.06 δ^36^Ar, ±0.1 for δ^38^Ar, ±0.8 for δO_2_/N_2_ (‰), ±0.9 for δO_2_/Ar (‰), ±0.56 for δCO_2_/Ar (‰).

For nitrite and nitrate analyses, water was filtered with a 0.45 μm (cellulose acetate membrane), poisoned with mercuric chloride and stored in 5 vials of 12 ml. Of which, 2 vials are treated with sulfamic acid to eliminate possible nitrite as NO volatile^84^. Nitrogen and oxygen isotopic compositions of nitrate and nitrites were analyzed by the denitrification method^85^. International reference materials (IAEA-N3, USGS-34 and USGS-35) and 5 in-house working standards were included within a batch of 96 bottles for a series of analysis. The N_2_O produced was then analyzed using a Thermo-Finnigan Delta V Plus. Analytical precisions were estimated to be within 0.3‰ for δ^15^N and δ^18^O.

## Supplementary information

Supplementary Figures.
