# Supplementary material for: Discovery of Afifi, the shallowest and southernmost brine pool reported in the Red Sea
Source: Sci Rep. 2020 Jan 22;10:910. doi: 10.1038/s41598-020-57416-w (PMC6976674; doi:10.1038/s41598-020-57416-w)
Supplement: Supplementary file 1 — Supplementary Figures. [file 41598_2020_57416_MOESM1_ESM.pdf]

Supplementary Information

Discovery of Afifi, the shallowest and southernmost brine pool reported in the Red Sea

Carlos M. Duarte[1], Anders Røstad[1], Grégoire Michoud[2], Alan Barozzi[2], Giuseppe Merlino[2], Antonio Delgado-Huertas[1,3], Brian C. Hession[4], Francis L. Mallon[4], Abdulakader M. Afifi[5], and Daniele Daffonchio[2]

[1] Red Sea Research Center (RSRC) and Computational Bioscience Research Center, King Abdullah University of Science and Technology (KAUST), Thuwal, 23955-6900, Saudi Arabia

[2] Red Sea Research Center (RSRC) and Biological and Environmental Science and Engineering Division (BESE), King Abdullah University of Science and Technology (KAUST), Thuwal, 23955-6900, Saudi Arabia

[3] Instituto Andaluz de Ciencias de la Tierra, CSIC-UGR, Avda. de las Palmeras 4, 18100 Armilla, Spain

[4] Coastal and Marine Resources Core Lab (CMOR), King Abdullah University of Science and Technology (KAUST), Thuwal, 23955-6900, Saudi Arabia

[5] Ali I. Al-Naimi Petroleum Engineering Research Center (ANPERC), Physical Science and Engineering Division (PSE), King Abdullah University of Science and Technology (KAUST), Thuwal, 23955-6900, Saudi Arabia

Figures Headings

Fig. S1: Red Sea Water Gating Strategy. A, B, C, D Negative control not stained and not filtered. E, F, G, H Negative control Sybr green stained and filtered (0.2 µm). I, J, K, L Red Sea Water. The cells count was performed by a combination of concatenated gates set on front scatter (FSC) and side scatter (SSC) G1, on SSC and Sybr Green height G2, on the Sybr Green height count G3, on the Sybr Green width and height G4. G1 and G2 were set to exclude the instrument noise (A, B, E, F, I, J). G3 was set to exclude the fluorescent events measured in the negative controls (C, G). We considered the events measured in the Red Sea water G3 as a total number of cells per sample (K). Events in G4 were considered a cluster of two cells and accounted twice (L).

Fig. S2: Brine Water Gating Strategy. A, B, C, D Negative control not stained and not filtered. E, F, G, H Negative control Sybr green stained and filtered (0.2 µm). I, J, K, L Brine Water. We performed the cells count with the same gating strategy explained in Fig. S1.

Fig. S1

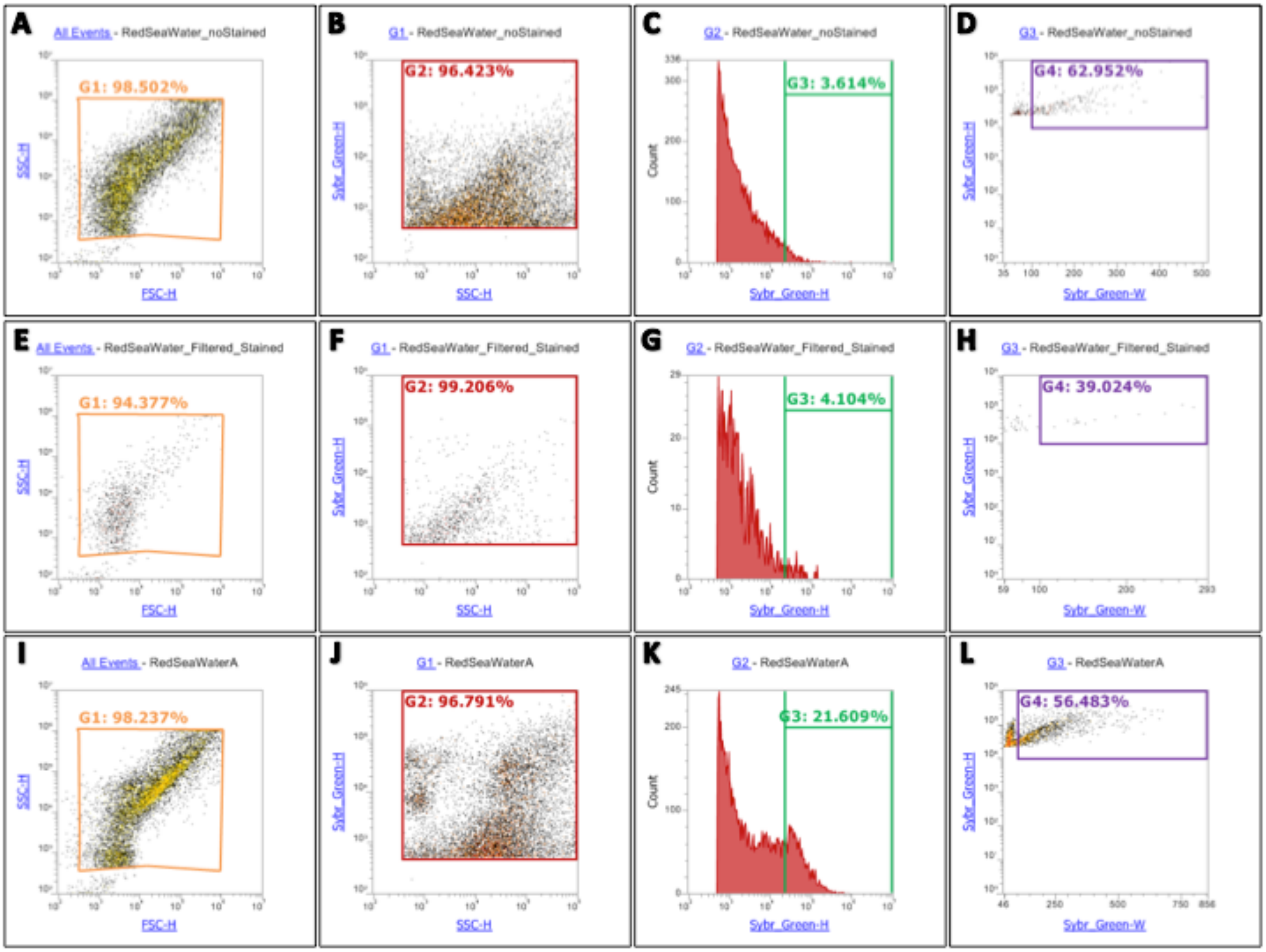

A
All Events - RedSeaWater_noStained
G1: 98.502%
SSC-H
FSC-H
B
G1 - RedSeaWater_noStained
G2: 96.423%
Sybr_Green-H
SSC-H
C
G2 - RedSeaWater_noStained
G3: 3.614%
Count
Sybr_Green-H
D
G3 - RedSeaWater_noStained
G4: 62.952%
Sybr_Green-H
Sybr_Green-W
E
All Events - RedSeaWater_Filtered_Stained
G1: 94.377%
SSC-H
FSC-H
F
G1 - RedSeaWater_Filtered_Stained
G2: 99.206%
Sybr_Green-H
SSC-H
G
G2 - RedSeaWater_Filtered_Stained
G3: 4.104%
Count
Sybr_Green-H
H
G3 - RedSeaWater_Filtered_Stained
G4: 39.024%
Sybr_Green-H
Sybr_Green-W
I
All Events - RedSeaWaterA
G1: 98.237%
SSC-H
FSC-H
J
G1 - RedSeaWaterA
G2: 96.791%
Sybr_Green-H
SSC-H
K
G2 - RedSeaWaterA
G3: 21.609%
Count
Sybr_Green-H
L
G3 - RedSeaWaterA
G4: 56.483%
Sybr_Green-H
Sybr_Green-W

Fig. S2

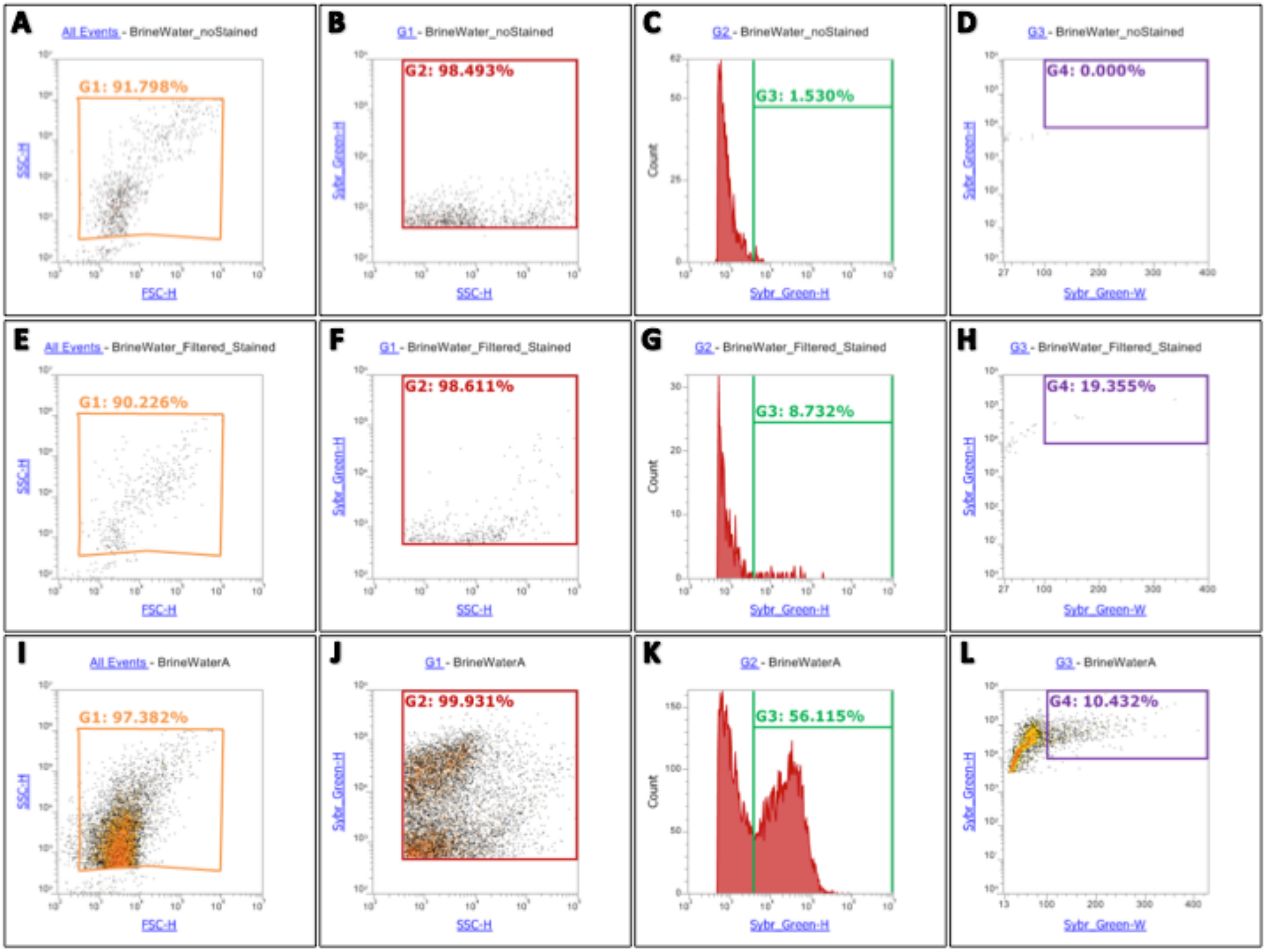
A
All Events - BrineWater_noStained
G1: 91.798%
SSC-H
FSC-H
B
G1 - BrineWater_noStained
G2: 98.493%
Sybr_Green-H
SSC-H
C
G2 - BrineWater_noStained
G3: 1.530%
Count
Sybr_Green-H
D
G3 - BrineWater_noStained
G4: 0.000%
Sybr_Green-H
Sybr_Green-W
E
All Events - BrineWater_Filtered_Stained
G1: 90.226%
SSC-H
FSC-H
F
G1 - BrineWater_Filtered_Stained
G2: 98.611%
Sybr_Green-H
SSC-H
G
G2 - BrineWater_Filtered_Stained
G3: 8.732%
Count
Sybr_Green-H
H
G3 - BrineWater_Filtered_Stained
G4: 19.355%
Sybr_Green-H
Sybr_Green-W
I
All Events - BrineWaterA
G1: 97.382%
SSC-H
FSC-H
J
G1 - BrineWaterA
G2: 99.931%
Sybr_Green-H
SSC-H
K
G2 - BrineWaterA
G3: 56.115%
Count
Sybr_Green-H
L
G3 - BrineWaterA
G4: 10.432%
Sybr_Green-H
Sybr_Green-W
